# Evaluation of topical methylene blue nanoemulsion for wound healing in diabetic mice

**DOI:** 10.1080/13880209.2023.2254341

**Published:** 2023-09-10

**Authors:** Yu Gao, Zhounan Jiang, Bin Xu, Ran Mo, Shiyan Li, Yanan Jiang, Demei Zhao, Wangbin Cao, Bin Chen, Meng Tian, Qian Tan

**Affiliations:** a Nanjing Drum Tower Hospital Clinical College of Nanjing Medical University, Nanjing, China; b The Second Affiliated Hospital, Zhejiang University School of Medicine, Hangzhou, China; c Hubei Xiangyang Central Hospital, Xiangyang, China; d Department of Burns and Plastic Surgery, Nanjing Drum Tower Hospital, the Affiliated Hospital of Nanjing University Medical School, Nanjing, China; e Nanjing Hospital of Traditional Chinese Medicine, Nanjing, China; f Institute of Plant Resources and Chemistry, Nanjing Research Institute for Comprehensive Utilization of Wild Plants, Nanjing, China; g Department of Plastic Surgery, Shanghai Fourth People’s Hospital, School of Medicine, Tongji University, Shanghai, China

**Keywords:** Diabetic wound, Nrf2, inhibit apoptosis

## Abstract

**Context:**

Diabetic wounds (DW) are a complication of diabetes and slow wound healing is the main manifestation. Methylene blue (MB) has been shown to exhibit therapeutic effects on diabetes-related diseases.

**Objective:**

To investigate the mechanisms of action of MB-nanoemulsion (NE) in the treatment of DW.

**Materials and methods:**

The concentration of MB-NE used in the *in vivo* and *in vitro* experiments was 0.1 mg/mL. Streptozocin-induced diabetic mice were used as models. The mice were separated into nondiabetic, diabetic, MB-NE treated, and NE-treated groups. Intervention of high glucose-induced human umbilical vein endothelial cells using MB-NE. The mechanism by which MB-NE promotes DW healing is investigated by combining histological analysis, immunofluorescence analysis, TUNEL and ROS assays and western blotting.

**Results:**

In diabetic mice, the MB-NE accelerated DW healing (*p* < 0.05), promoted the expression of endothelial cell markers (α-SMA, CD31 and VEGF) (*p* < 0.05), and reduced TUNEL levels. *In vitro*, MB accelerated the migration rate of cells (*p* < 0.05); promoted the expression of CD31, VEGF, anti-apoptotic protein Bcl2 (*p* < 0.05) and decreased the expression of the pro-apoptotic proteins cleaved caspase-3 and Bax (*p* < 0.05). MB upregulated the expression of Nrf2, catalase, HO-1 and SOD2 (*p* < 0.05). In addition, MB reduced the immunofluorescence intensity of TUNEL and ROS in cells and reduced apoptosis. The therapeutic effect of MB was attenuated after treatment with an Nrf2 inhibitor (ML385).

**Discussion and conclusion:**

This study provides a foundation for the application of MB-NE in the treatment of DW.

## Introduction

Dysregulated wound healing in patients with diabetes is an increasing health concern worldwide that poses a high risk of amputation and presents a mortality rate similar to that in some cancers (Martins-Mendes et al. 2014). Factors affecting diabetic wound healing are complex and involve impaired cell proliferation and migration, uncontrolled inflammatory responses, impaired angiogenesis and the disturbed secretion of multiple cell growth factors (Holl et al. 2021). Therefore, further studies on therapies that promote diabetic wound healing are required.

Methylene blue (MB), a cationic thiazine dye, has demonstrated therapeutic efficacy in various diabetes-related diseases, including diabetic retinopathy (Hao et al. 2019), diabetic vascular changes (Privistirescu et al. 2018) and diabetic heart disease (Berthiaume et al. 2017). MB modulates the function of vascular endothelial cells (Privistirescu et al. 2018), and recent studies suggest that it accelerates wound healing in both animal models and human tissues (Cesar et al. 2022). Nanoemulsions (NEs) are metastable systems with internal phase droplets smaller than 1000 nm and are generally 200–600 nm in size. This dosage form is obtained by dispersing water in oil (or oil-in-water emulsions) and then achieving stabilization using surfactants (Singh et al. 2017). Because of their extremely small droplet size, high surface tension and large surface area, NEs are beneficial for the efficient delivery of active compounds through the skin. These properties allow the uniform distribution of droplets on the surface of the skin and facilitate the penetration of active compounds into the skin (Shakeel et al. 2012). NEs can be formed spontaneously via simple preparation processes that exhibit slow release and targeting effects, thereby improving drug solubility, reducing drug enzymatic degradation *in vivo* and improving bioavailability; hence, they have been used widely as a drug carrier (Moghassemi et al. 2022).

In this study, we develop an effective MB-NE for the treatment of diabetic wounds. We determine the properties of MB-NE and investigate its mechanism of action in diabetic wound healing. We perform *in vivo* studies using C57/B6 mice to further investigate the role of the MB-NE in wound healing. Specifically, we investigate the effects of MB on human umbilical vein endothelial cells (HUVECs) *in vitro*.

## Materials and methods

### Materials

Methylene blue was purchased from Yuanye Biotechnology (Shanghai, China). Isopropyl myristate (IPM) was obtained from Yuanye Biotechnology (Shanghai, China). Hydrogenated castor oil (CO40) was purchased from Dow Chemicals (Midland, MI). Polyethylene glycol 400 (PEG 400) was obtained from Yuanye Biotechnology (Shanghai, China). Streptozocin (STZ) was purchased from Macklin Biochemicals (Shanghai, China). All other reagents were of analytical grade.

### Preparation and characterization of MB-NE

To prepare blank NEs or MB-NEs, we used CO40 as the surfactant, PEG400 as the co-surfactant, IPM as the oil phase and ultrapure water as the aqueous phase. First, we prepared CO40 and PEG400 as emulsifiers at a ratio of 2:1. Subsequently, we added MB to the oil phase of the IPM at a ratio of 1:4 and dissolved it completely using a mechanical stirrer (Farmoudeh et al. 2020). The oil phase containing MB was added to the emulsifier at a ratio of 1:9. The aqueous phase was deposited into the oil phase under magnetic stirring, and the NE was prepared by stirring for 60 min. The MB-NE was prepared at a concentration of 0.1 mg/mL. A transmission microscope image was used to observe the NE morphology, and an appropriate amount of MB-NE was obtained and diluted for 5-6 min. The excess liquid was blotted off with filter paper, and 1% phosphotungstic acid solution (pH 7.0) was added for staining. The particle size, polydispersity index (PDI) and zeta potential of the NEs were measured at room temperature using a laser particle size analyzer (ZanoZS90, UK).

### Animal experiment

All animal experiments were approved by the Animal Ethics Committee of First Nanjing Hospital, which is affiliated with Nanjing Medical University (DWSY-23010314). Male C57/B6 mice (weight 19–23 g, 6–8 weeks old) were purchased from Jiangsu Jizui Pharmachem Biotechnology. Before the experiment, all mice were kept in a suitable environment for one week, temperature 24 ± 2 °C, humidity 55 ± 5%, 12 h light/dark cycle, allowed free access to standard laboratory water and food. The mice were separated into four groups (*n* = 8) as follows: Group I, nondiabetic mice (NC); Group II, diabetic mice (DM); Group III: Methylene blue nanoemulsion-treated mice (MB-NE) (0.1 mg/mL) and Group IV: Blank nanoemulsion-treated mice (NE) (0.1 mg/mL). Glycosuria was induced via an intraperitoneal injection of STZ (50 mg/kg) in all three groups of mice, except for the NC group, which was injected with an equal volume of saline. Random blood glucose measurements were performed on the third day after STZ injection, followed by random blood glucose measurements at 3-day intervals. If the random blood glucose is ≥ 16.7 mmol/L for more than two consecutive measurements and the mice show signs of diabetes such as lethargy, depression, lustreless hair and excessive urination, food and drink, then they are regarded as successful diabetic models. Two weeks after STZ injection, full-thickness skin excision wounds were created in the mice. A full-thickness skin excision wound of the same size (diameter, 1 cm) was created on the backs of all mice. The MB-NE group of MB-NE and NE groups were treated daily with MB-NEs (0.1 mg/mL) and blank NEs, respectively. MB-NEs or blank NEs were applied evenly to the wounds of mice, covering the entire wound as appropriate. The wounds of all mice were photographed on days 0, 3, 7 and 14 after healing. The wound images were analyzed using the ImageJ software.

### Histological analysis and immunofluorescence

On day 14, all mice were anesthetized by intraperitoneal injection of 1% sodium pentobarbital (45 mg/kg), and mice were sacrificed. Mouse skin tissues were harvested, fixed with 4% paraformaldehyde, and embedded in paraffin. Paraffin sections measuring 5 μm thick were stained with haematoxylin and eosin (H&E) and Masson’s trichrome. For immunofluorescence staining, skin tissues were embedded in an optimal cutting temperature (OCT) compound (Thermo Fisher Scientific, Waltham, MA). Cryopreserved skin sections (5 μm thick) were stained with primary antibodies against CD31 (550274; BD Biosciences, Franklin Lakes, NJ) and SMA (ab15580; Abcam, Cambridge, UK), followed by staining with specific fluorescent secondary antibodies (Thermo Fisher Scientific). The nuclei were stained with DAPI (Santa Cruz Biotechnology, Santa Cruz, CA). Images were captured using a Leica DM5500 microscope (Leica Microsystems, Wetzlar, Germany) and analyzed using the ImageJ software.

### Cell culture and treatment

HUVECs (National Collection of Authenticated Cell Cultures, Shanghai, China) were used for *in vitro* experiments. The HUVECs were cultured in Dulbecco’s modified Eagle medium (DMEM; 2.5 g/L or 4.5 g/L D-glucose, BasalMedia Technologies Co.). The medium contained 10% foetal bovine serum (Gibco, Green Island, NY) and 1% penicillin/streptomycin (cat. no. C0224; Beyotime, Shanghai, China) and was incubated at 37 °C at 5% CO_2_. MB was dissolved in PBS to obtain different concentrations of MB solutions. DMEM at a concentration of 2.5 g/L was used for the control group. The HUVECs were treated with MB, high glucose HG (4.5 g/L) or HG + MB for 24 h in a suitable culture environment. In addition, the HUVECs were treated with ML385 (10 μM) 2 h before treatment with the MB solution.

### Cell viability (CCK-8 assay)

The effects of different concentrations of MB solution on the activity of HUVECs were assessed using the CCK-8 assay. A 96-well plate (5000 cells/well) was used and filled with different concentrations (0, 50, 100, 250, 500 and 1000 μM) of MB solution. Twenty-four hours after the addition of the MB solution, 10 μL of CCK-8 solution was added to the wells and the optical density was measured at 450 nm after 1 h of incubation. The measurements were repeated three-times, and then data analysis was performed. On the basis of the statistical results, the concentration of the MB solution with the highest cell activity was obtained and used in subsequent experiments to intervene with the HUVECs.

### Scratch assay

HUVECs were seeded in six-well plates. The number of inoculated cells was maintained as uniformly as possible, and an overnight inoculation was performed to achieve a 100% fusion rate. The next day, using a sterile 200-μL pipette tip, a scratch was created on the cell layer perpendicular to the cell plane. Subsequently, unadhered cells were washed with PBS, and the initial width of the scratch was photographed using a microscope. The cells were incubated at 37 °C and 5% CO_2_ for 24 h. The width of the scratch was photographed using a microscope, and the images were analyzed using the ImageJ software.

### TUNEL staining

The extent of apoptosis in the wound tissues and HUVECs was assessed using the TUNEL assay. TUNEL staining of tissue sections and HUVECs was performed using a TUNEL assay kit (Beyotime, China) based on the manufacturer’s instructions. First, the tissues and cells were permeabilized with 0.3% Triton X 100. Subsequently, they were incubated overnight with the primary antibodies and for 1 h with the corresponding secondary antibodies. Staining of cell nuclei was performed using DAPI. Finally, their images were captured under a fluorescence microscope (Olympus, Tokyo, Japan), which were then analyzed using the ImageJ software.

### Immunofluorescence analysis of HUVECs

The HUVECs were transferred to 24-well plates (8000 cells/well), which were maintained under basal culture conditions with different or no intervention conditions for 24 h. Subsequently, the HUVECs were fixed with 4% paraformaldehyde and blocked using donkey serum for 30 min at 37 °C. The Nrf2 (12721; 1:200; Cell Signaling Technology) primary antibody was added to the wells and then allowed to stand overnight at 4 °C. After washing the HUVECs with PBS, specific secondary antibodies were added to them and incubated for 60 min at 37 °C protected from light, and DAPI staining was performed. Finally, the cells were observed under a fluorescence microscope (Olympus, Tokyo, Japan).

### Analysis of oxidative stress

The ROS levels in the HUVECs were detected using a ROS assay kit (CA1410; Solarbio Science, Beijing, China). After washing the cells with PBS, they were incubated with 10 μm DCFH-DA for 20 min at 37 °C. After incubation, the cells were washed with PBS and photographed using a fluorescence microscope (Olympus, Tokyo, Japan).

### Western blotting (WB)

The wound tissues or treated HUVECs were lysed using RIPA buffer (Thermo Scientific) containing a protease inhibitor (Roche, Switzerland) and 1% phosphatase inhibitor (Sigma, Long Island, NY). Protein was quantified using a BCA protein assay kit (Beyotime, Jiangsu, China). Equal amounts of protein were separated using SDS-PAGE and then transferred to polyvinylidene difluoride membranes (Millipore, Burlington, MA). The cells were closed with 5% skim milk for 1 h and then washed with TBST before they were added with the following primary antibodies: CD31 (A2104; 1:1000; ABclonal), α-SMA (14395-1-AP; 1:1000; Proteintech), VEGF (ab46154; 1:1000; Abcam, Cambridge, UK), β-actin (AC038; 1:10000; ABclonal), cleaved caspase-3 (9664; 1:1000; Cell Signaling Technology), Bcl2 (ab182858; 1:2000; Abcam), Bax (ab182733; 1:2000; Abcam), Nrf2 (12721; 1:1000; Cell Signaling Technology), HO-1 (ab68477; 1:1000; Abcam), catalase (A11220, 1:1000; ABclonal), and SOD2 (A19576, 1:1000; ABclonal). The cells were incubated overnight at 4 °C with diluted primary antibodies. After washing with TBST, the membranes were incubated with a horseradish peroxidase-conjugated secondary antibody (SA00001-2; Proteintech) for 1 h at room temperature. Finally, the bands were detected using a multifunctional imaging system (Tanon 5200, Shanghai, China) with an ECL kit (P1010, Applygen, Beijing, China) and the images were analyzed using the ImageJ software.

### Statistical analysis

The data of this study were presented as mean ± SEM and analyzed via one-way ANOVA. Subsequently, Tukey’s *post hoc* test was performed for multiple comparisons using the SPSS 22.0 software. *p* < 0.05 was considered statistically different.

## Results

### Characterization of MB-NE

**Figure 1.**
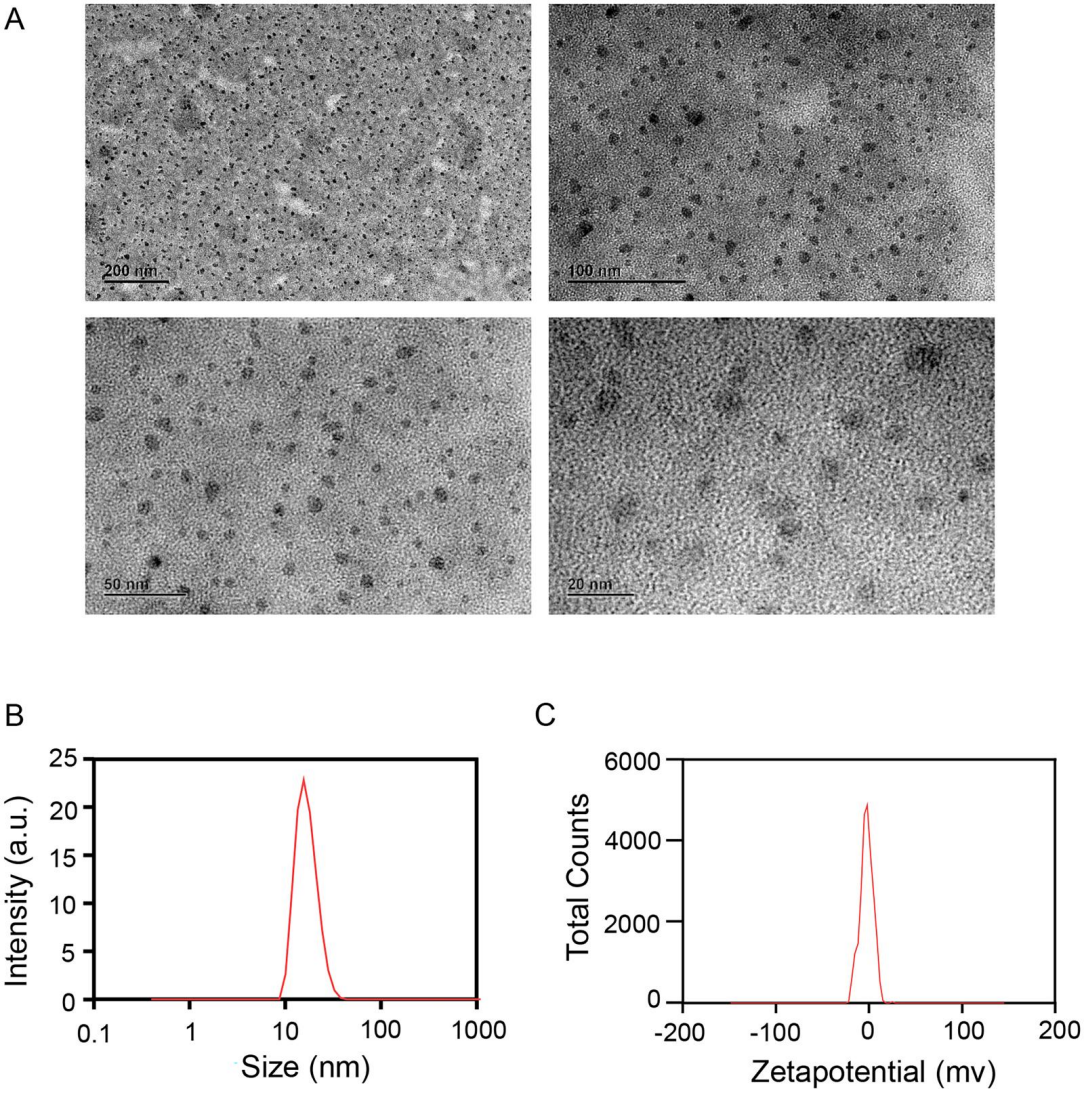
Preparation and characterization of MB-NE. (A) Transmission microscope image (TEM) of MB-NE. (B) Particle size distribution of MB-NE. (C) Zeta potential of MB-NE (MB, methylene blue; NE, nanoemulsion).

The droplet size must be emphasized because our goal is to establish an NE. By definition, particle size is a key indicator of NEs and a visual indicator of the emulsion-forming performance of NEs, which should be less than 100 nm, as evidenced by our results. On the basis of transmission electron microscopy images, the MB-NE droplets exhibited a rounded spherical structure with uniform size and good formability (Figure 1(A)). The particle size of the MB-NE was 20.88 ± 0.12 nm, which was within the particle size range of NEs, thus indicating that the prepared NEs had a small particle size. The PDI indicates the degree of uniform distribution of droplets, whose value is typically between 0 and 1; the smaller the PDI value, the better the dispersion uniformity. A PDI index of less than 0.3 typically signifies a dispersion of good uniformity. In addition, a PDI of 0.236 ± 0.12 (Figure 1(B)) indicates a relatively uniform distribution of NE droplets with high stability, which can extend the drug release time and improve the drug efficacy. The zeta potential was −2.45 ± 0.65 mV (Figure 1(C)), indicating that the surface of the NE had a certain number of negative charges. An appropriate number of charges causes less aggregation and sedimentation between particles, which is conducive to increasing the stability of the system and affords high physical stability. The results show that the prepared NE satisfied the basic requirements of NEs.

### Topical application of MB-NE promotes wound healing in diabetic mice

**Figure 2.**
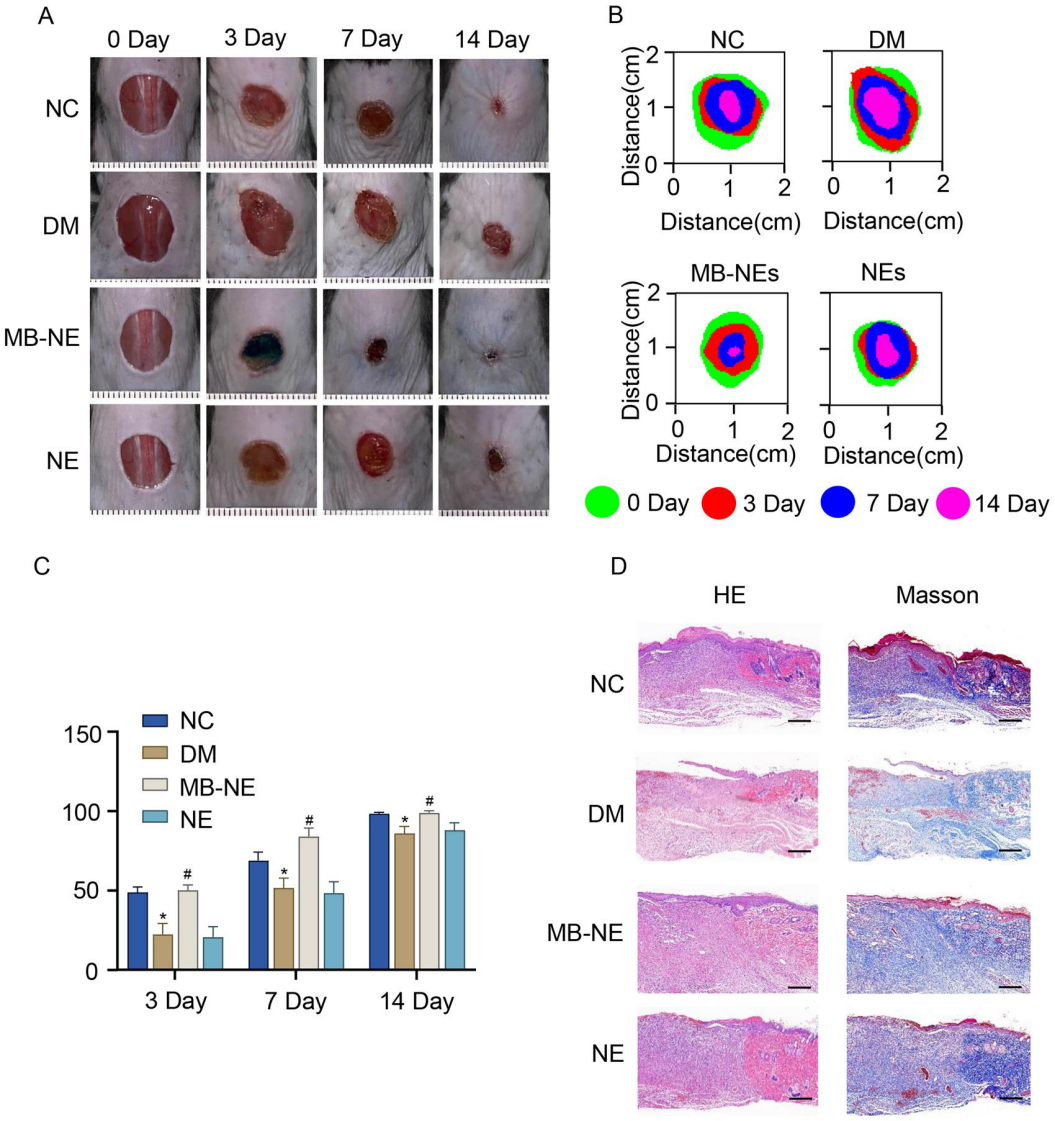
MB-NE promotes wound healing in diabetic mice. (A) Representative wound images of each group of mice at different time points. (B) Wound extent simulations of each group of mice at different time points. (C) Wound healing rate of each group of mice at different time points. (D) HE and Masson staining of wound skin tissue sections of each group of mice on day 14 (*n* = 5). (E) Wound length analysis (*n* = 5). Scale bar: 1 mm. NC, control group; DM, diabetic group; MB-NE, diabetic + methylene blue nanoemulsion group; NE, diabetic + blank nanoemulsion group. *p < 0.05, vs. NC, ^#^*p* < 0.05, vs. DM.

To investigate the effect of the MB-NE on diabetic wound healing, full-thickness wounds were created on the backs of normal and STZ-induced diabetic mice. As shown in Figure 2(A–C), the wound healing rate of the DM group was lower than that of the NC group beginning from day 3 following injury (*p* < 0.05, vs. NC), and the topical application of the MB-NE significantly promoted the healing of diabetic wounds (*p* < 0.05, vs. DM). Furthermore, histological examinations of periwound tissues were conducted to assess the healing quality. The DM group showed a thinner epidermis, less collagen deposition and a slower re-epithelialization process than the NC group. Meanwhile, MB-NE treatment, but not NE treatment, significantly promoted collagen deposition and re-epithelialization in diabetic mice (Figure 2(D–E)) (Shen et al. 2021). These results suggest that the MB-NE promotes wound healing in diabetic mice.

### MB-NE promotes angiogenesis and reduces apoptosis in diabetic mice

**Figure 3.**
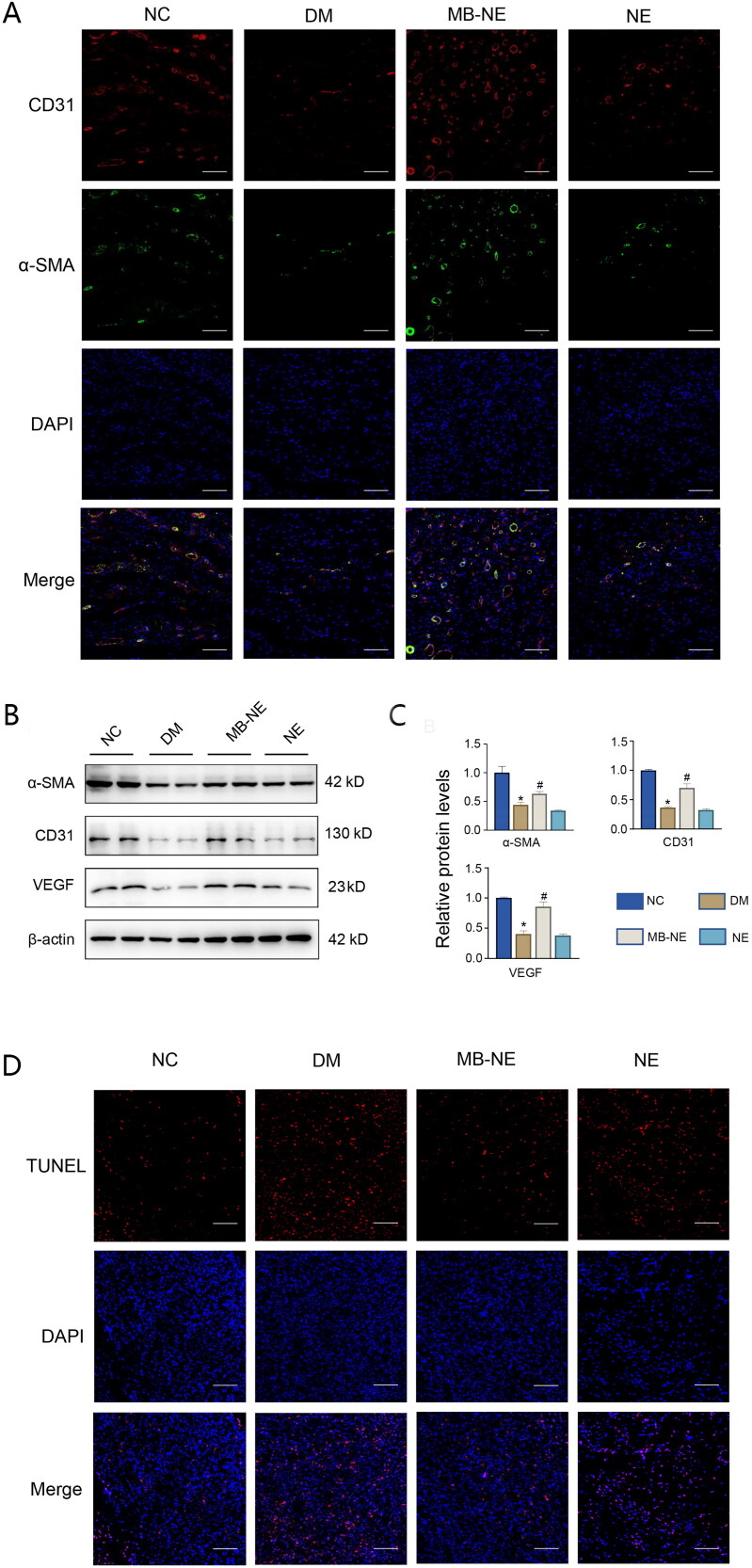
Pro-angiogenic and anti-apoptotic effects of MB-NE on diabetic mouse wounds. (A, B) Western blotting and quantitative analysis of α-SMA, VEGF and CD31 in wound tissue of diabetic mice. (C) Expression of CD31 and α-SMA in wound detected via immunofluorescence staining. Scale bar: 100 μm. (D) TUNEL staining showing apoptosis in each group. scale bar: 100 μm. NC, control group; DM, diabetic group; MB-NE, diabetic group + methylene blue nanoemulsion; NE, diabetic + blank nanoemulsion group. *p < 0.05, vs. NC, ^#^*p* < 0.05, vs. DM.

To investigate the effect of the MB-NE on angiogenesis during wound healing, we performed immunofluorescence to observe endothelial cell markers CD31 and -SMA (Figure 3(A)). The DM group showed lower CD31 and -SMA levels than the NC group. Increased numbers of α-SMA and CD31-positive endothelial cells were observed in the MB-NE-treated wounds compared with the observation in the DM group. The protein expression of CD31, α-SMA and VEGF in the skin wound tissue of the different groups was assessed using WB (Figure 3(B,C)). Consistent with the immunofluorescence results, the levels of CD31, α-SMA and VEGF increased significantly in the MB-NE group compared with those in the DM group (*p* < 0.05). TUNEL assays were performed to observe apoptosis, which is considered positive if the nuclei are stained red. The number of apoptotic cells in the DM group was significantly higher than that in the NC group, and the MB-NE administration significantly reduced apoptosis (Figure 3(D)). The results show that MB-NE accelerated wound healing in diabetic mice by promoting angiogenesis and reducing apoptosis.

### MB promotes angiogenesis and inhibits apoptosis in vitro

**Figure 4.**
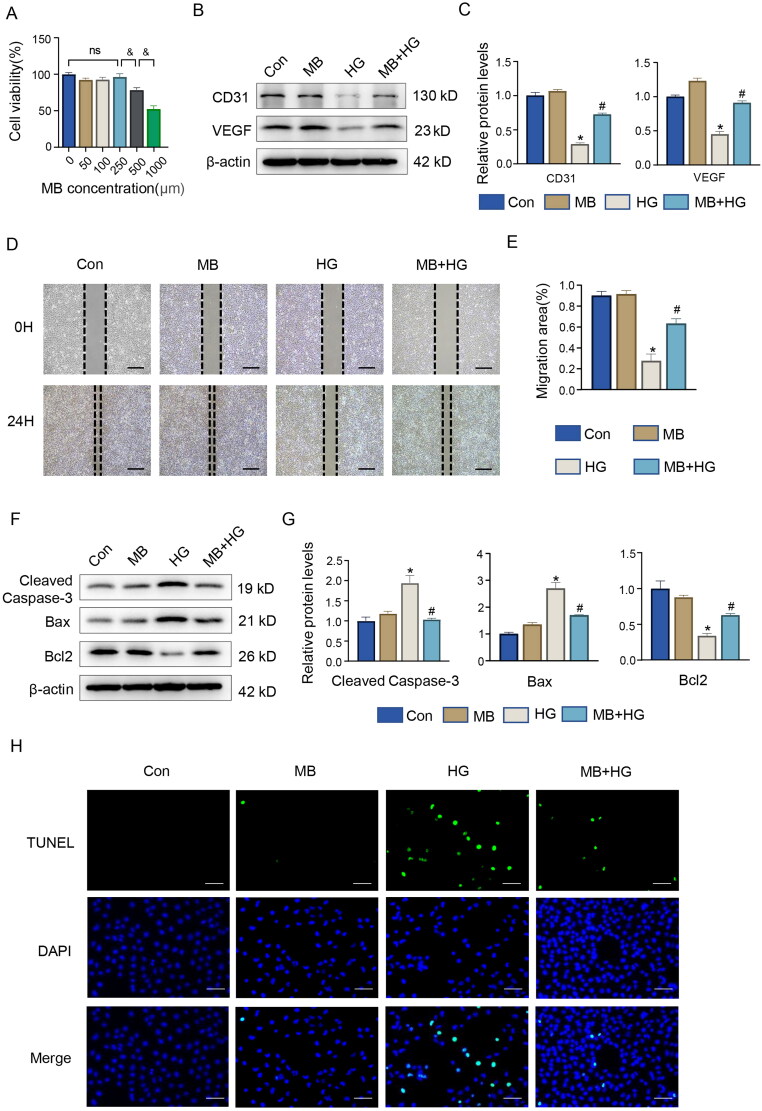
MB promoted angiogenesis and alleviated HG-induced apoptosis in HUVECs. (A) Effect of different concentrations of MB solution on cell activity. Confirmation of 250 μM as concentration of MB solution used in subsequent experiments. (B, C) Western blotting and quantitative analysis of CD31 and VEGF in HUVECs. (D, E) Scratch assay in different groups and change in results. Scale bar: 100 μm. (F, G) Western blotting and quantitative analysis of cleaved caspase-3, Bax and Bcl2 in HUVECs. (H) TUNEL staining showing apoptosis in each group. Scale bar: 100 μm. Con: control group; MB: methylene blue group; HG: high glucose group; MB + HG: high glucose + methylene blue group. &*p* < 0.05, vs. 250 μm. *p < 0.05, vs. Con. ^#^*p* < 0.05, vs. HG. HUVECs, human umbilical vein endothelial cells.

To further investigate the mechanism of action of MB, we performed *in vitro* experiments using HUVECs. On the basis of CCK-8 analysis, a concentration of 250 μM MB was shown to impose the least prominent effect on cell activity (Figure 4(A)) and hence used for the intervention. The effect of MB on HG-induced expressions of angiogenesis-related proteins was determined via WB. The results show that HG inhibited the expressions of CD31 and VEGF (*p* < 0.05, vs. Con), which were reversed by MB (*p* < 0.05, vs. HG) (Figure 4(B,C)). Figure 4(D,E) show the results of cell scratching, where the rate of scratch recovery was significantly slower in the HG-treated HUVECs (*p* < 0.05, vs. Con), whereas HG + MB recovered better than HG (*p* < 0.05, vs. HG).

In addition, the effect of MB treatment on the expression of HG-induced apoptosis-related proteins in the HUVECs was determined via WB. The results show that HG increased the expression levels of the pro-apoptotic proteins Bax and cleaved-caspase-3, as well as decreased the expression of anti-apoptotic protein Bcl2, which was effectively ameliorated via MB treatment (Figure 4(F,G)). The immunofluorescence staining of TUNEL showed that HG-induced apoptosis and MB exerted an anti-apoptotic effect (Figure 4(H)). Thus, MB promotes diabetic wound healing, which is associated with angiogenesis promotion and apoptosis inhibition.

### MB reduces oxidative stress by activating Nrf2 and downstream signalling in vitro

**Figure 5.**
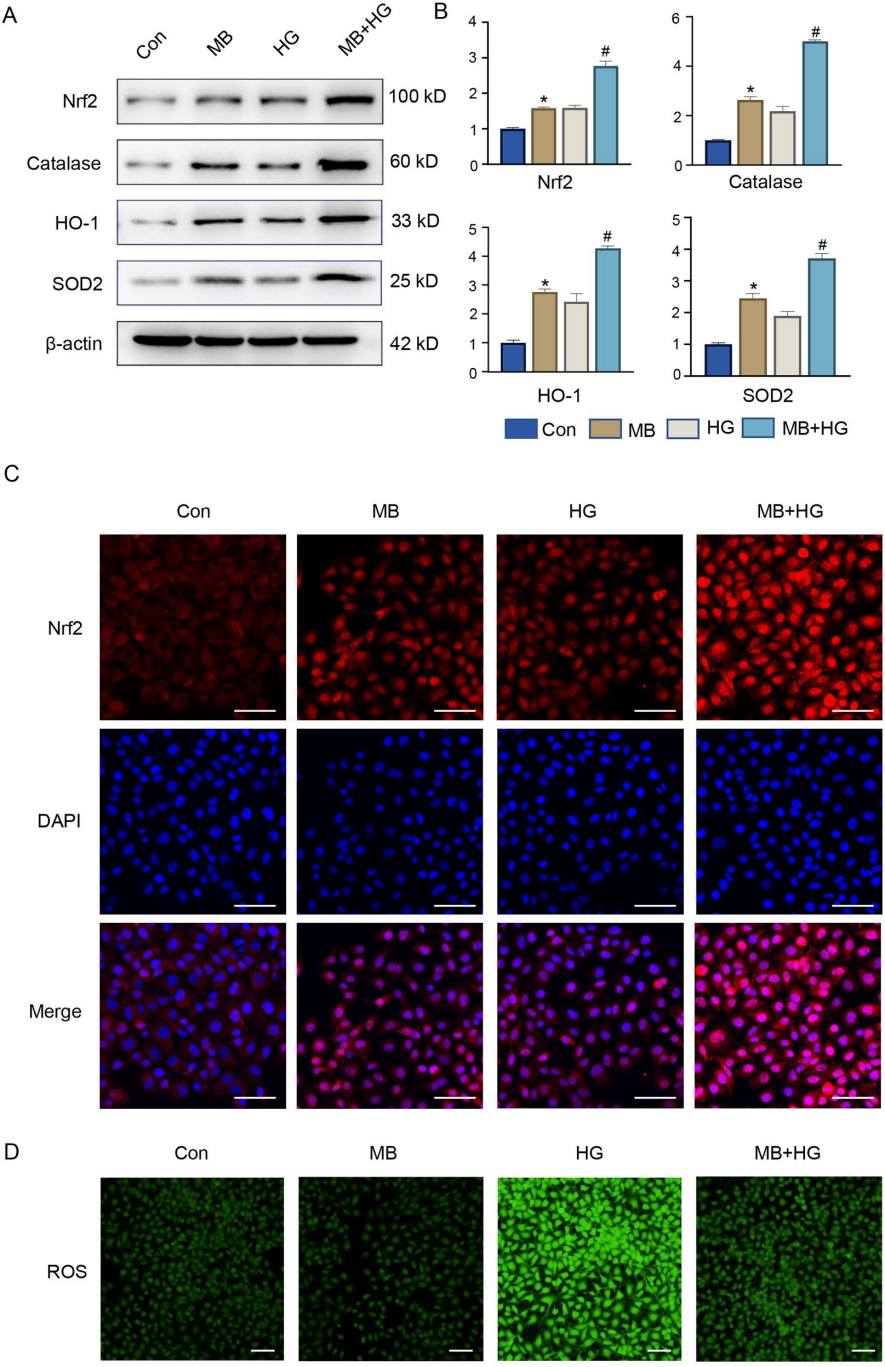
MB reduced HG-induced oxidative stress by activating Nrf2 signalling in HUVECs. (A, B) Western blotting and quantitative analysis of Nrf2, catalase, HO-1 and SOD2 in HUVECs. (C) Expression of Nrf2 detected via immunofluorescence staining. Scale bar: 100 μm. (D) ROS level detected via immunofluorescence staining. Scale bar: 100 μm. Con: control group; MB: methylene blue group; HG: high glucose group; MB + HG: high glucose + methylene blue group. *p < 0.05, vs. Con. ^#^*p* < 0.05, vs. HG.

To further understand the molecular mechanisms underlying MB in diabetic wounds, we investigated Nrf2 and its downstream signalling pathways. The WB results show that the expressions of Nrf2, catalase, HO-1 and SOD2 elevated in the MB- and HG-treated HUVECs (Figure 5(A,B)). The use of MB under HG conditions further elevated the expression of these proteins (*p* < 0.05, vs. HG), indicating that MB can exert antioxidant stress and demonstrate a higher activation of Nrf2 along its downstream signals. The results of the immunofluorescence analysis were consistent with those of WB (Figure 5(C)). In the MB group, the same trend as in the HG group was observed. The increase shown in the MB group is attributable to cell stimulation by MB, which increases the expression of related proteins. The increase in the HG group may be due to compensatory changes in the cells induced by high glucose levels. In addition, results of immunofluorescence analysis show that HG increased the ROS levels, which decreased MB was used (Figure 5(D)).

### Nrf2 is a key target for MB to promote diabetic wound healing

**Figure 6.**
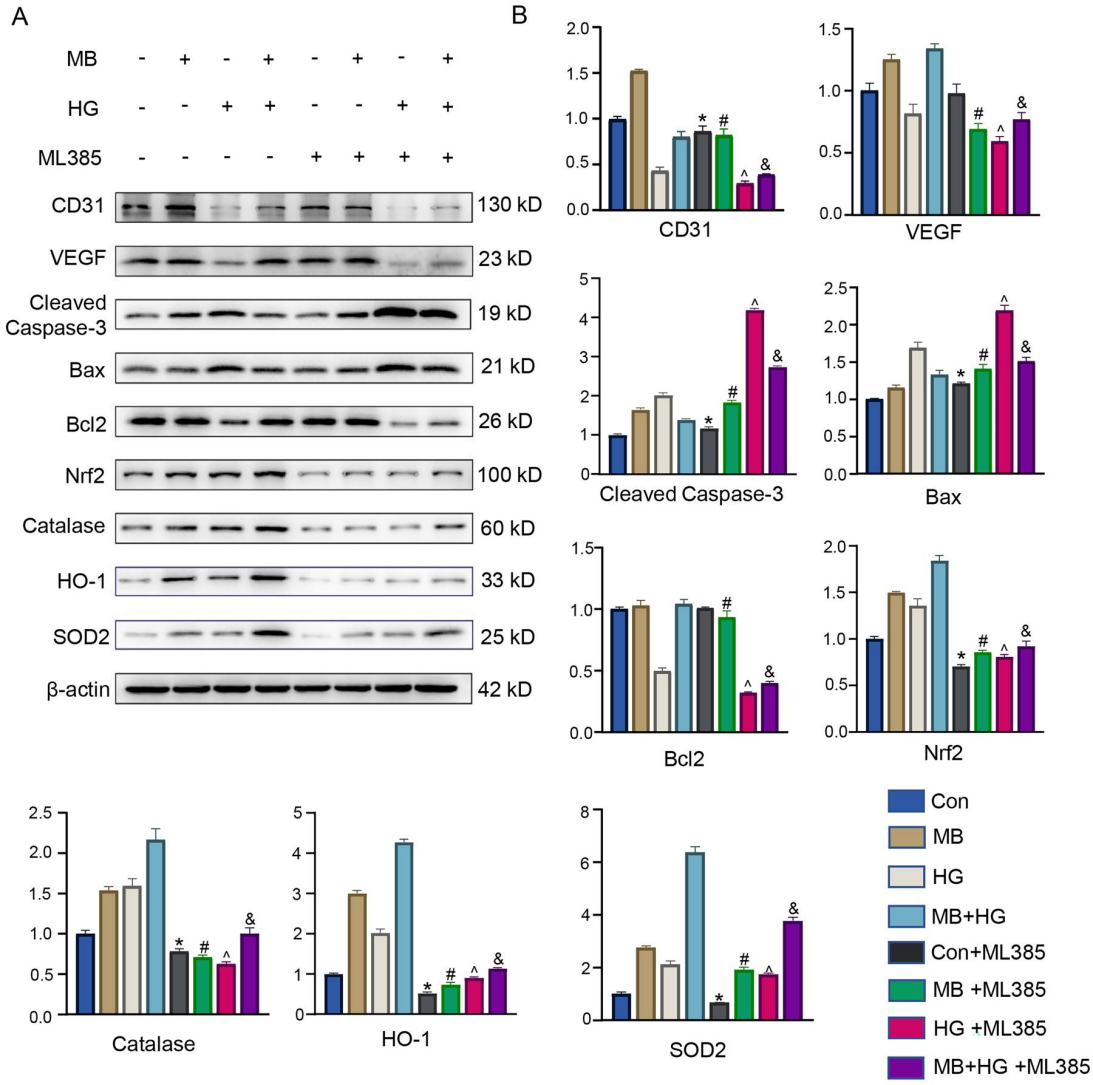
Inhibition of Nrf2 attenuated protective effect of MB on HUVECs. (A, B) Western blotting and quantitative analysis of each protein in different groups. (C) ROS level detected via immunofluorescence staining. (D) TUNEL staining showing apoptosis in each group. Scale bar: 100 μm. Con: control group; HG: high glucose group; HG + MB: high glucose + methylene blue group; HG + MB + ML385: high glucose + methylene blue group + ML385. *p < 0.05, vs. Con. ^#^*p* < 0.05, vs. MB. ^ *p* < 0.05, vs. HG. ^&^*p* < 0.05, vs. MB + HG.

To investigate the mechanism of action of MB, we used an Nrf2 inhibitor (ML385) to observe the downstream signal expression levels. The WB results show that the expression of proteins related to angiogenesis, apoptosis and oxidative stress was significantly affected by ML385 (Figure 6(A,B)). The proteins associated with angiogenesis were CD31 and VEGF, and their expression decreased significantly in cases with ML385 as compared with the cases without ML385 (*p* < 0.05). The proteins associated with apoptosis were cleaved caspase-3, Bax and Bcl2. After ML385 was used, the expression of cleaved caspase-3 and Bax upregulated significantly as compared with that for the case without ML385 (*p* < 0.05); meanwhile, the expression of Bcl2 downregulated in all groups except the control group, where no significant difference was indicated (*p* < 0.05). The proteins associated with oxidative stress were Nrf2, catalase, HO-1 and SOD2, all of which showed a significant decrease in expression after treatment with ML385 compared with the case without ML385 (*p* < 0.05). Furthermore, the results of TUNEL immunofluorescence staining show that MB exerted an anti-apoptotic effect, which was inhibited by ML385. MB reduced HG-induced ROS production, which was reversed by ML385 treatment. These results suggest that the inhibition of Nrf2 expression affects the expression of other related proteins and attenuates the therapeutic effect of MB. MB is crucial in promoting angiogenesis, inhibiting apoptosis and reducing oxidative stress through the Nrf2 signalling pathway.

## Discussion

This study develops a novel preparation method for MB and evaluates its potential effects on diabetic wound healing. We synthesized an MB-NE based on the size of the NEs, which accelerated wound healing in diabetic mice by promoting angiogenesis, inhibiting apoptosis and reducing oxidative stress. In addition, the therapeutic effect of MB was reversed considerably via treatment with an Nrf2 inhibitor. These results suggest that MB-NEs may exert therapeutic effects through the Nrf2 signalling pathway, thus providing new insights into the treatment of diabetic wounds.

MB was first synthesized in 1876 and can be used in a diverse range of applications with few side effects (Tucker et al. 2018). It has been used for treating various diseases such as methemoglobinaemia, malaria, vascular paralysis, infectious shock and Alzheimer’s disease (Atamna and Kumar 2010; Paciullo et al. 2010; Schirmer et al. 2011). MB permeates easily through biological membranes, soluble in water and organic solvents, and can enter various cellular organelles, such as mitochondria, lysosomes and nuclei (Rohs and Sklenar 2004; Atamna et al. 2008; Wu et al. 2009). The potential of MB in promoting wound healing has been investigated (Farmoudeh et al. 2020; Perez et al. 2021). Composite antimicrobial foam dressings containing MB and gentian violet have been reported to accelerate the recovery of chronic wounds of the lower extremities by reducing pain and allowing a higher bacterial load on the wound surface (Coutts et al. 2014). The incorporation of MB into composite nanofibers in a diabetic wound mouse model showed wound healing promotion through re-epithelialization, collagen deposition, elevated CD34 and TGF-β expression, and decreased CD95+ expression (Abdel Khalek et al. 2021). As high-performance delivery agents, NEs are characterized by their high kinetic stability, extended-release profiles, improved therapeutic efficiency and good dermal permeability (Moghassemi et al. 2022). The application of curcumin-NEs to diabetic rat wounds significantly downregulated oxidative stress and enhanced collagen deposition, thus accelerating skin tissue regeneration (Ali et al. 2022). In addition, topical administration of linezolid-NEs and NEs containing boron and/or zinc promoted wound healing in the treatment of diabetic wounds (Gundogdu et al. 2022; Haider et al. 2022).

Diabetic wounds, which do not heal quickly, complicate clinical treatment and endanger the physical and mental health of patients. Diabetic wound healing involves several processes, including cell-to-cell chemotaxis, cell proliferation, vascular neogenesis and extracellular matrix deposition (Lee et al. 2001). Wound healing is typically impaired in patients with diabetes owing to vascular dysfunction, high oxidative stress, neurohypoxia, impaired angiogenesis, defective collagen deposition and weak immune response (Kamar et al. 2019). Neovascularization at the wound site facilitates the formation of granulation tissue and prolongs the residence time of keratinocytes, thus facilitating diabetic wound healing (De Bock et al. 2013). Previous studies based on three-dimensional skin tissue models showed that using MB promoted wound healing by accelerating fibroblast migration and proliferation as well as improved skin thickness and hydration (Xiong et al. 2017). Consistent with previous results, our current results indicate that MB-NEs promoted angiogenesis near the wound by upregulating α-SMA, VEGF and CD31. VEGF promotes endothelial cell proliferation (Kahroba and Davatgaran-Taghipour 2020), and the interaction between CD31 and VEGF promotes angiogenesis (Martin and Leibovich 2005). However, in another study pertaining to the retina, MB prevented VEGF upregulation in response to retinal damage, which suggested the preventive anti-angiogenic effect of MB (Fernández et al. 2020). Therefore, the effects of MB on angiogenesis may differ from those observed in models and should be investigated further.

Programmed cell death is a key factor for maintaining normal cell development and tissue homeostasis in multicellular organisms (Arya et al. 2014). Increasing evidence shows that excessive apoptosis is the leading cause of delayed diabetic wound healing (Lao et al. 2019). Anti-apoptotic effects are crucial in the recovery of diabetic wounds. MB has been shown to improve the integrity of the blood–brain barrier and reduce the apoptosis of neuronal cells during traumatic brain injury (Shen et al. 2019). MB inhibits apoptosis by stabilizing the mitochondria and limiting the permeability of the outer mitochondrial membrane, thus preventing the release of pro-apoptotic molecules. In addition, MB inhibits the p53-Bax-Bcl2-caspase-3 cascade, which consequently inhibits the apoptotic signalling pathway and exerts anti-apoptotic effects (Jiang et al. 2015). In this study, MB inhibited apoptosis by promoting the expression of Bcl2 and inhibiting the expression of cleaved caspase-3 and Bax.

Sustained oxidative stress impairs diabetic wound healing by damaging cellular proteins, lipids and DNA, thereby inducing tissue dysfunction (Long et al. 2016). On the basis of the results obtained using the diabetes model in this study, more severe oxidative stress occurred with higher expressions of oxidative stress-related proteins, including Nrf2, catalase, HO-1 and SOD2, which can be attenuated via treatment with MB. Recently, MB was shown to exhibit antioxidant properties that protect cells from oxidative stress under pathological conditions by functioning as an alternative receptor for electrons during tissue oxidation and competitively inhibiting the reduction of molecular oxygen to superoxides (Salaris et al. 1991; Beldi et al. 2021). In diabetes, high ROS levels impair wound healing, whereas vascular neogenesis occurs in environments with low levels of ROS (Schafer and Werner 2008). On the basis of skin burn models, MB treatment can effectively reduce the progression of necrosis in wound tissue and offers antioxidant effects, which reduce oxidative stress at the wound site (Rosique et al. 2017). On the basis of the results of anti-aging studies, MB can bypass the activity of complex I/III, thereby reducing ROS production (Tretter et al. 2014; Svab et al. 2021). Furthermore, MB functions as an alternative electron carrier in the mitochondrial respiratory chain, thus enhancing mitochondrial activity while reducing oxidative stress (Tucker et al. 2018). In addition, low-dose MB treatment reduces ROS production (Poteet et al. 2012).

Nrf2 serves as a master regulator of oxidative stress and is a central conductor of cellular defence. At the cellular or organismal level, physical and chemical injuries involving oxidative stress activate Nrf2 transcription factors, which consequently induces the expression of various cytoprotective genes (Chen and Maltagliati 2018). In the present study, MB promoted diabetic wound healing through the Nrf2 signalling pathway, whereas ML385 attenuated the therapeutic effect of MB. Thus, the Nrf2 signalling pathway is key in promoting diabetic wound healing and may be a target of MB. *In vitro* experiments using rat macrophages showed that high glucose inhibited Nrf2 activation, induced oxidative stress and suppressed the expression of antioxidant-related genes (Li et al. 2019). In addition, researchers demonstrated that bioactive compounds promoted diabetic wound healing through Nrf2-related pathways, such as resveratrol (Zhou et al. 2021), curcumin (Senger and Cao 2016) and Prussian blue nanoparticles (Xu et al. 2022) (Choi et al. 2014). Pharmacological activators of Nrf2 have been shown to resist oxidative stress and promote wound healing in diabetic foot ulcer (Victor et al. 2020).

## Conclusions

We successfully constructed an MB-NE and applied it for the treatment of diabetic wounds. MB possesses therapeutic effects in promoting angiogenesis, inhibiting apoptosis and reducing oxidative stress by activating the Nrf2 signalling pathway, thus accelerating diabetic wound healing. This study provides a foundation for the application of MB-NEs in the treatment of diabetic wounds.

## Data Availability

The data that support the findings of this study are available from the corresponding author QT upon reasonable request.
